# The Use of Arbuscular Mycorrhizal Fungi to Improve Strawberry Production in Coir Substrate

**DOI:** 10.3389/fpls.2016.01237

**Published:** 2016-08-19

**Authors:** Louisa Robinson Boyer, Wei Feng, Natallia Gulbis, Klara Hajdu, Richard J. Harrison, Peter Jeffries, Xiangming Xu

**Affiliations:** ^1^NIAB-EMREast Malling, UK; ^2^Plantworks Ltd.Sittingbourne, UK; ^3^School of Biosciences, University of KentCanterbury, UK

**Keywords:** strawberry, yield, growing substrate, AMF, coir, Class I yield quality

## Abstract

Strawberry is an important fruit crop within the UK. To reduce the impact of soil-borne diseases and extend the production season, more than half of the UK strawberry production is now in substrate (predominantly coir) under protection. Substrates such as coir are usually depleted of microbes including arbuscular mycorrhizal fungi (AMF) and consequently the introduction of beneficial microbes is likely to benefit commercial cropping systems. Inoculating strawberry plants in substrate other than coir has been shown to increase plants tolerance to soil-borne pathogens and water stress. We carried out studies to investigate whether AMF could improve strawberry production in coir under low nitrogen input and regulated deficit irrigation. Application of AMF led to an appreciable increase in the size and number of class I fruit, especially under either deficient irrigation or low nitrogen input condition. However, root length colonization by AMF was reduced in strawberry grown in coir compared to soil and Terragreen. Furthermore, the appearance of AMF colonizing strawberry and maize roots grown in coir showed some physical differences from the structure in colonized roots in soil and Terragreen: the colonization structure appeared to be more compact and smaller in coir.

## Introduction

Strawberry is an important horticultural crop in the UK and is a highly nutritious and important food source. Strawberry accounted for 67% of all soft fruit production worth an estimated £247 million in 2013 (DEFRA, 2015), and this is set to rise significantly over the coming years. Recently, a significant trend in commercial strawberry cropping has been to move away from traditional field cultivation toward production into substrate. Industry estimates that more than 50% of the UK strawberry production is produced in substrates, usually coir (coconut fiber) and mostly under protection (polythene tunnel or glasshouse). This change was intended to mitigate the threat of soil-borne fungal pathogens, principally wilt (*Verticillium dahliae* Kleb). Chemical treatments have been an indispensable tool for controlling soil-borne pathogens; however, several of these treatments are already banned or face an uncertain future due to legislation (Martin, 2003). There are many significant benefits to the adoption of substrates in commercial strawberry cropping, such as to extension of the growing season, increased ease of picking and better control of the crop from fertigation and pollination regimes. However, this practice relies heavily on high inputs of water and fertigation; these inputs are estimated to be more than doubled compared to a field grown crop, with an increased cost of up to £1800 per Hectare.

Arbuscular mycorrhizal fungi (AMF) penetrate the roots of plants to form a mutualistic symbiotic relationship. Mineral nutrients, mainly phosphorus, nitrogen and water are extracted from the soil via the extensive hyphal network and transferred to the plant. Organic carbon compounds are transferred to the AMF in return. They are known to improve plant nutrient uptake, protect plants from pathogens (Borowicz, 2001; Ismail and Hijri, 2012; Ren et al., 2013) and buffer against adverse environmental conditions, especially drought (Smith et al., 2010; Robinson-Boyer et al., 2015). A number of studies have reported the beneficial effects of mycorrhiza on strawberry plants (Castellanos-Morales et al., 2010) and commercial AMF inoculum has been shown to increase both growth (crowns, roots and leaf area) and tolerance to water stress in micro-propagated strawberry (Borkowska, 2002).

The maintenance of a developed and diverse population of AMF and other soil micro-organisms is important in achieving sustainable agriculture (Jeffries et al., 2003) thus reducing the requirement of such high levels of fertigation. However, products containing AMF are rarely used in commercial agriculture because of (a) difficulties in producing AMF inoculum in large quantities, (b) their variable beneficial effects, and (c) uncertainties in the benefits with added AMF in the presence of resident AMF populations. Substrates such as coir are usually devoid of beneficial microbes such as AMF; thus introducing them into substrate production is more likely to generate benefits.

This paper reports results from three studies on the use of AMF in strawberry production in coir substrate. First, we assessed whether use of AMF in substrate could improve strawberry fruit yield in respect to water stress and nutrient input. This work showed positive effects of the addition of AMF in fruit production despite observing low levels of AMF colonization and compact, immature mycorrhizal structures inside colonized roots of strawberry. Thus, we conducted further experiments to better understand the extent and structure of root colonization in different types of substrate (including soil). Furthermore, to establish if the effects observed on root colonization were limited to strawberry only, we included maize in the experiment as maize is a common, highly mycorrhizal host plant of AMF.

## Materials and methods

### Effect of AMF inoculation of strawberry in coir

**Figure 1:**
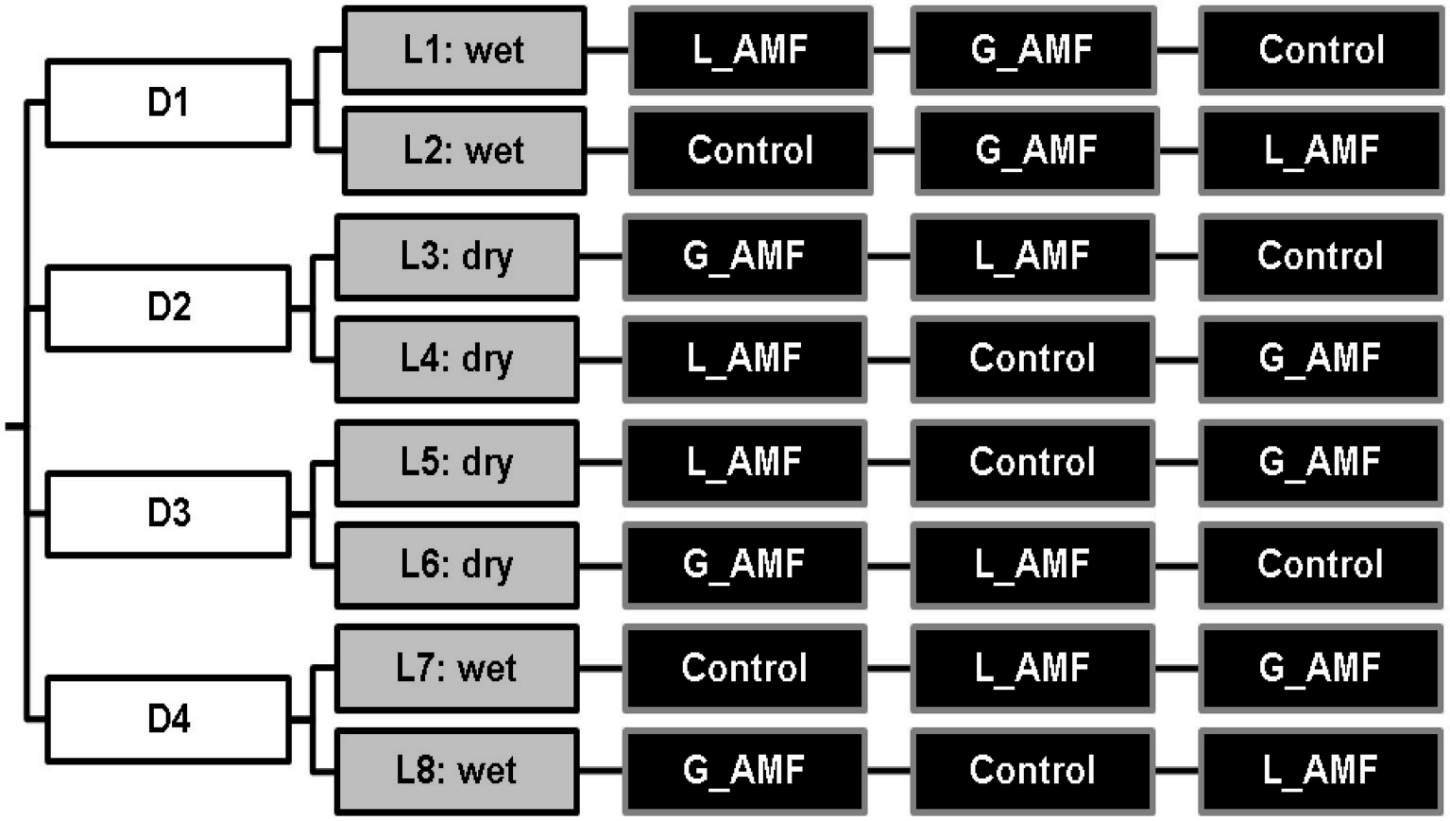
**Experimental setup for the study of the effect of AMF inoculation on strawberry in coir**. AMF inoculation treatments consisted of either non-inoculated control, Liquid spore suspension of *R. irregularis* (L_AMF) or granular application of commercial inoculum (G_AMF). Two levels of irrigation were included, fully watered (wet), and 60% RDI (dry), along with 2 levels of Nitrogen level, D1 and 2 at the standard commercial rate and D3 and 4 at a 60% reduction of the standard rate. The experiment was repeated three times.

The experimental design was a full factorial design with three factors: AMF inoculation, irrigation and nutrient. For AMF inoculation, there were three treatment levels: negative control with no inoculum added, and application of either granular or liquid formulation of AMF (G_AMF or L_AMF). There were two irrigation regimes: well-watered, to capacity (WW), and regulated deficit irrigation (RDI, 60% of the WW). There were two nutrient input regimes: standard or reduced nitrogen input (60% of the standard). Thus, there were 12 treatments in total (see Figure 1). This experiment was conducted on three separate occasions, with two replicates of each treatment each time.

Agronomical management of strawberry (including dosatron setting, nutrient composition and irrigation) followed previously established protocols (Xu et al., 2013), which were based on current commercial practices. From a combination of visual assessment of water leakage from coir bags and moisture content measurements, estimated using a Delta-T “WET” sensor (Delta-T Devices, Cambridge, UK), the amount of irrigation water was adjusted as necessary via a Galcon irrigation timer. Overall, the volume of irrigation water applied increased gradually over time, reached the maximum at the first week of blossom and thereafter remained at this level, equivalent to 2 L per day per bag.

Irrigation and fertigation were delivered to plants via eight irrigation lines using drippers, two of which were controlled by a separate irrigation controller (dosatron). Each dosatron was randomly allocated to one of the four nutrient and water combinational treatments. Within each irrigation line, there were three replicate bags, each allocated to one of the three AMF treatments. Thus, the experiment was a split plot design—the main plot was the dosatron (two irrigation lines) and the subplot was the individual coir bag. All coir bags (BotaniCoir, England) prior to planting were saturated with water over a period of 2 weeks in order to re-hydrate the coir. Inoculum of AMF was supplied by PlantWorks Ltd, Kent, UK, the granular formulation applied as commercially available “Rootgrow” (*Funneliformis mosseae, F. geosporus, Claroideoglomus claroideum, Glomus microagregatum, Rhizophagus irregularis*), containing propagules of spores, hyphal and root fragments. The liquid application was an *in-vitro* produced preparation of *R. irregularis* DAOM197198 (consisting of sterile water and spores).

Cold-stored (−2°C) runners of cv. Elsanta (Hargreaves Plants, UK) were planted in coir bags. At the time of planting, for the G_AMF treatment, 20 g of granular AMF was placed to a single planting hole before the plant was planted; for L_AMF, a liquid AMF suspension [4 ml estimated to be taken up per plant] was applied to the roots of individual runners and for the control nothing was added. For both G_AMF and L_AMF each plant received ca. 6650 propagules of AMF estimated using MPN analysis (Cochran, 1950). After the onset of flowering a mini hive of bees, *Bombus terrestris*, (Agralan, UK) was introduced to the compartment to pollinate (with the exception of the first replicate experiment). Plants were grown in a GroDome compartment (Unigro, UK) set at 22°C day/20°C night with a 16 h day/8 h night cycle with supplementary lighting.

A sample of roots from a number of plants was assessed prior to planting to check for colonization by AMF. Roots were cleared with KOH before being stained using Trypan Blue and assessed microscopically for root length colonization (RLC) using the grid-line intersect method (McGonigle et al., 1990). Colonization was expressed as a percentage of the root colonized by AMF. Ripe fruit were picked regularly (2–3 times weekly). Except for the first experiment, fruit were divided by size into Class I (above 18 mm diameter) & II and weighed separately for individual bags and the number of fruit was recorded. For the first experiment, because of smaller fruit (lack of pollinators), fruit were not divided into Class I or II. After harvesting, fresh weight of individual plants (both above- and below-ground parts) was determined. A composite sample of roots was taken for each coir bag at harvest to check colonization by AMF. Only fresh, recently formed roots were sampled, and the original runner roots were avoided. Roots were stained and assessed as above.

### The effect of substrate on colonization by AMF

#### Substrate effect and time of inoculation

Maize (cv Thalys, Cotswold Seeds, UK) was used in this experiment since it is known to be highly responsive to AMF and a common host for production of commercial AMF and is used here to study the effect of substrate on root colonization. There were three treatment factors: pre-emergence inoculation with AMF (PreAMF: Yes or No), post-emergence inoculation with AMF (PostAMF: Yes or NO). Substrates compared were Top soil: S, Terragreen: T, coir: C, and peat-free compost: PF. These substrates are commonly used for commercial cropping with the exception of Terragreen (attapulgite clay; OilDry, UK) which is routinely used in the study of AMF, giving a clear indication of “expected” colonization. A randomized block design was used with five blocks. There were two pots per treatment in each block: one for destructive sampling 4 weeks after transplanting, and the other after 10 weeks.

Maize seeds were first soaked in sterile water for 24 h. Multi-cell trays (cell volume 250 ml) were filled with Terragreen. Half of the cells were amended with 10 g of granular AMF inoculum to allow inoculation of seedlings (pre-emergence). One seed was manually sown 2 cm deep per cell. Seedlings for the Pre-AMF inoculation treatment were sampled and checked microscopically for colonization by AMF (as above) and only those seedlings with colonization were retained. Prior to transplanting, a sufficient number of coir bags were thoroughly wetted; coir from these bags was then used to fill pots. Similarly, top soil (ca top 10–15 cm) from a plot at East Malling Research was obtained to fill pots; peat-free compost was purchased commercially (Dobbies, UK). Seedlings were transplanted approximately 2 weeks after sowing. On the day before transplanting, all pots were thoroughly watered to reach the fully-wet state. A planting hole was made in each pot and 10 ml AMF sprinkled into the hole for those pots allocated to the Post-AMF inoculation treatment. Then a single seedling was transplanted to each single pot (4 L). All plants were fed with Vitafeed 102 (Vitax, UK) 1 gL^−1^ every 2 weeks. The height and stem diameter, just above the substrate surface and colonization by AMF (RLC) were assessed destructively 4 and 10 weeks after transplanting. The root samples analyzed at 10 weeks were sampled from two positions on the plants, firstly very close to the inoculation site and secondly from the peripheral roots, and stained and assessed as above.

#### Effect of coir substrate on AMF colonization

For this study, both maize and strawberry were used to compare colonization by AMF in coir and in Terragreen. Maize seedlings (cv. Jubilee F1, B&Q, UK) were obtained as in the previous experiment, except Terragreen was not amended with inoculum of AMF. Strawberry module plants (cv. Elsanta), produced from tipping in compost, were obtained from a commercial nursery (Hargreaves plants, UK); plants derived in this way have shown in previous work to be free from colonization by AMF (Xu, unpublished data), although a few plants were tested prior to the experiment to confirm this. Individual plants were transplanted to 1 L pots [one plant per pot]; all plants were inoculated with 20 g of granular inoculum of AMF at the time of transplanting. There were 10 replicate pots per treatment (substrate [Terragreen or coir] and host [strawberry or maize]). A complete randomisation design was used. A standard commercial fertigation scheme of N-P-K = 120-45-176 for strawberry was used to manage the plants (J. Atwood, ADAS, England, per. comm.). Only eight strawberry and four maize plants per substrate (randomly selected from 10 plants in each treatment) were sampled to assess root colonization 10 weeks after transplanting; the amount of vesicles, arbuscules and hyphae were also recorded.

### Data analysis

ANOVA of a split-plot design was applied to strawberry data from all three experiments treating individual experiments as a blocking factor, using GenStat 13 (VSN International, England). In addition to total Class I yield and number of Class I fruit, average individual fruit weight was also analyzed. Fresh plant weight was also used as a co-variate in ANOVA but it did not alter the main results; therefore, only results from ANOVA without the co-variate are presented. Interactions between three factors: AMF, irrigation and nitrogen input were statistically tested. For the data on AMF colonization in different substrates, standard ANOVA were used to compare treatment effect. In all analyses, once ANOVA indicated significant effects of a specific treatment factor or interaction, pairwise comparison was then carried out based on the LSD test. Common diagnostic plots (e.g., q-q plot, residuals-fitted value plot) did not reveal apparent violation of the normality and homoscedasticity assumptions. Hence no transformation was necessary in order to satisfy ANOVA assumptions for fruit yield and RLC data.

## Results

### AMF on strawberry production in coir

**Figure 2:**
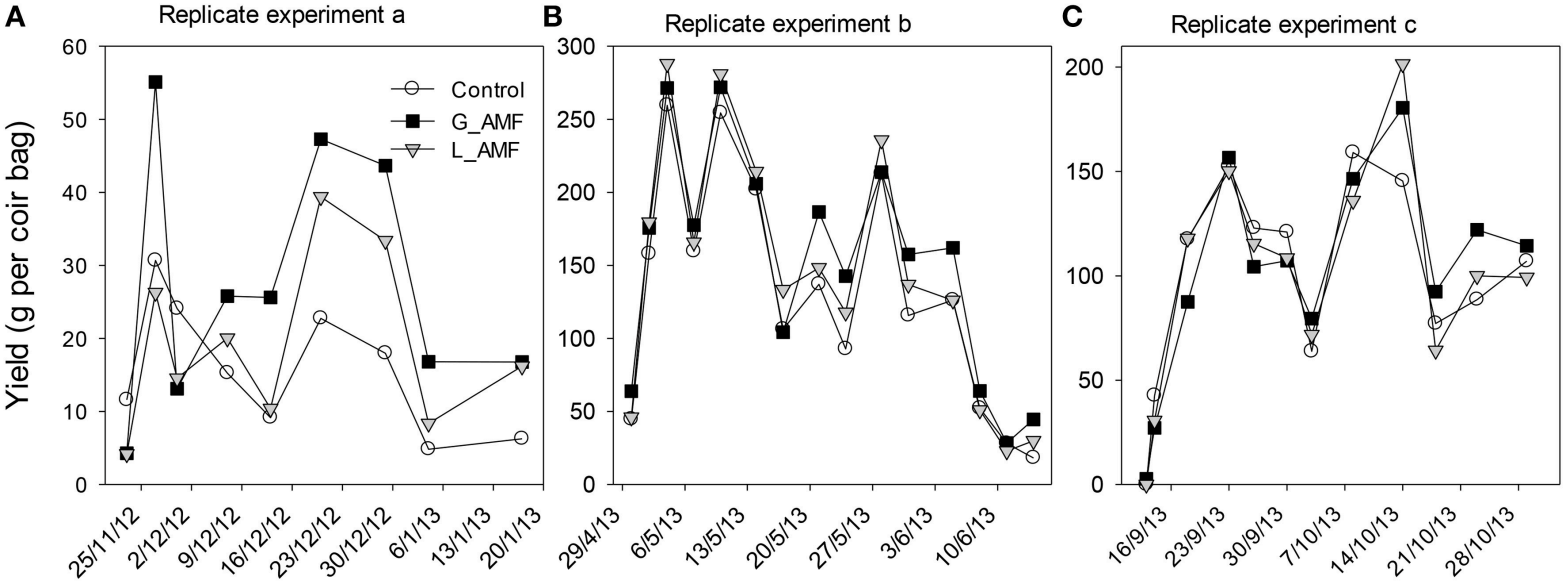
**Class I fruit yield (g per coir bag) for each pick date for the three replicate experiments (A–C)**. Each point was the average of eight individual bags over the four combinations of nitrogen and irrigation treatments. Yield from the first experiment was much lower than the other two because bees were not provided for pollinations.

Strawberry plants in all treatments grew normally and there were no visual differences in plant growth between treatments. Fewer and lighter fruit were produced in the first replicate experiment than in the other two experiments, due to less developed fruit from the lack of insect pollination. Plants in each coir bag on average produced 57, 127, and 108 fruit for the 1^st^, 2^nd^, and 3^rd^ replicate experiment, respectively; the corresponding average fruit weight was 4.7, 16.8, and 11.3 g. There was a large variation in fruit yield among individual picks in all three replicate experiments but the three AMF treatments followed a similar trend over time (Figure 2). AMF-treated strawberries (particularly G_AMF) had increased fruit production in the mid to late harvest period (Figure 2).

**Figure 3:**
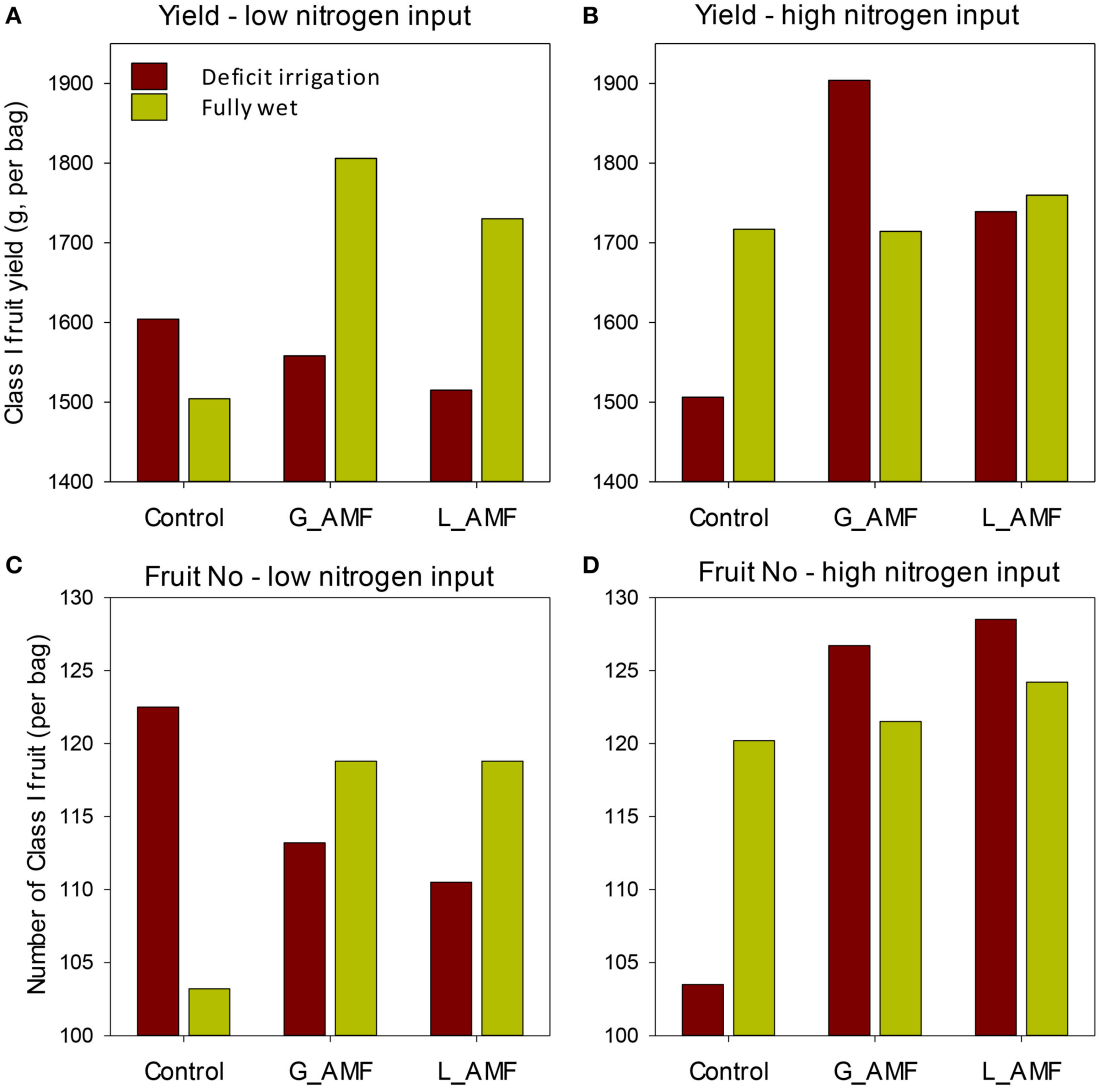
**Average class I fruit yield (A,B) and number of fruit (C,D) for each combination of AMF, nitrogen input and irrigation treatments for strawberry grown in coir bags in the replicate experiments**. The standard error of differences [sed] for the main AMF treatments was 63.0 g and 3.6 for yield and fruit number, respectively; the corresponding value for irrigation and nitrogen was 83.2 g and 7.2. The sed for the means of each AMF, irrigation and nitrogen combination was 156.3 g [yield] and 11.7 [fruit number], except when comparing means with the same combination of irrigation or nitrogen—which were 126 g and 7.3, respectively.

Because of the lack of bee pollinators, the 1st replicate experiment was excluded from statistical comparisons, hence all the subsequent presentations were from statistical analysis of replicate experiments 2 and 3 (Table 1). There were significant differences in the yield [total class I fruit weight; *F*_(2, 24)_ = 3.43, *P* < 0.05], and number of fruit per plant [*F*_(2, 24)_ = 3.30, *P* < 0.05] among AMF treatments. The G_AMF treatment led to higher (*P* < 0.05) yields than the control but not different from the L_AMF treatment (Figure 3). There were also no significant differences in yields between the L_AMF and the control treatments. On average the G_AMF and L_AMF treatments led to a greater (*P* < 0.05) number of fruit than the control (Figure 3). In both experiments, G_AMF had higher yield than L_AMF, although this difference is not statistically significant. Higher yields and more fruit were obtained in both high nitrogen and well-watered treatments than the low nitrogen and RDI treatments but these differences were not statistically significant (Table 1). For both the average fruit weight and plant fresh weight, none of treatments resulted in significant differences (Table 1).

**Table 1:** **Summary of ANOVA (*F*-values) of strawberry class I yield in two replicate experiments where granular and liquid AMF products were applied to strawberry plants grown in coir bags**.

**Terms**	**Degree of freedom**	**Yield**	**Fruit number**	**Fruit weight**	**Plant weight**
Experiment stratum	1	125.96	7.23	201.37	0.58
**EXPERIMENT × LINE STRATUM**					
Irrigation	1	0.66	0.00	1.88	0.63
Nitrogen	1	1.56	0.78	0.01	1.97
Irrigation × Nitrogen	1	0.41	0.09	1.89	0.08
Residual	11				
**EXPERIMENT × LINE × BAG STRATUM**					
AMF	2	3.43^*^	3.30^*^	1.41	1.89
AMF × Irrigation	2	0.27	0.10	0.50	0.81
AMF × Nitrogen	2	0.2	1.7	1.22	0.36
AMF × Irrigation × Nitrogen	2	4.59^*^	7.45^**^	1.52	0.09
Residual	24				

Plants were also subjected to two irrigation (high, low) and two nitrogen (high, low) treatments, delivered through automated fertigation pipe lines. A split-plot design was used in which the fertigation line and individual coir bags were the main and sub-plots, respectively.

*, ** Significant at the level of 0.05 and 0.01, respectively.

All interactions involving any two factors were not statistically significant (Table 1). However, the three-way interaction was significant for both the total yield [*F*_(2, 24)_ = 4.59, *P* < 0.05] and average number of fruit per plant [*F*_(2, 24)_ = 7.45, *P* < 0.01]. The interactions mainly resulted from the fact that the increase in the yield and number of fruit associated with AMF application was for the high nitrogen input under the deficit irrigation but the low nitrogen input under the wet treatment (Figure 3).

Prior to planting, runners were colonized by AMF and the RLC ranged from 20 to 40% for all three experiments. However, after the final harvest, there was almost no colonization (average < 1%) found in roots of the control, non-inoculated, plants and the level of AMF colonization found in the roots of the treated plants was low (average < 15%) and highly variable among samples; many samples failed to show any colonization. There were no differences between treatments in RLC.

### The effect of substrate on colonization by AMF

#### The effect of substrate on RLC of maize

There was no AMF colonization in non-inoculated plants when assessed 4 or 10 weeks after transplanting. For inoculated plants, RLC at 4 weeks ranged from 0 to 75.0% (with an average of 25.3%); only for five plants were AMF not observed. At 10 weeks, average AMF colonization over all roots, regardless of the position of sampled roots in relation to the inoculation site, was 49.2%; only for a single plant were AMF not observed.

**Figure 4:**
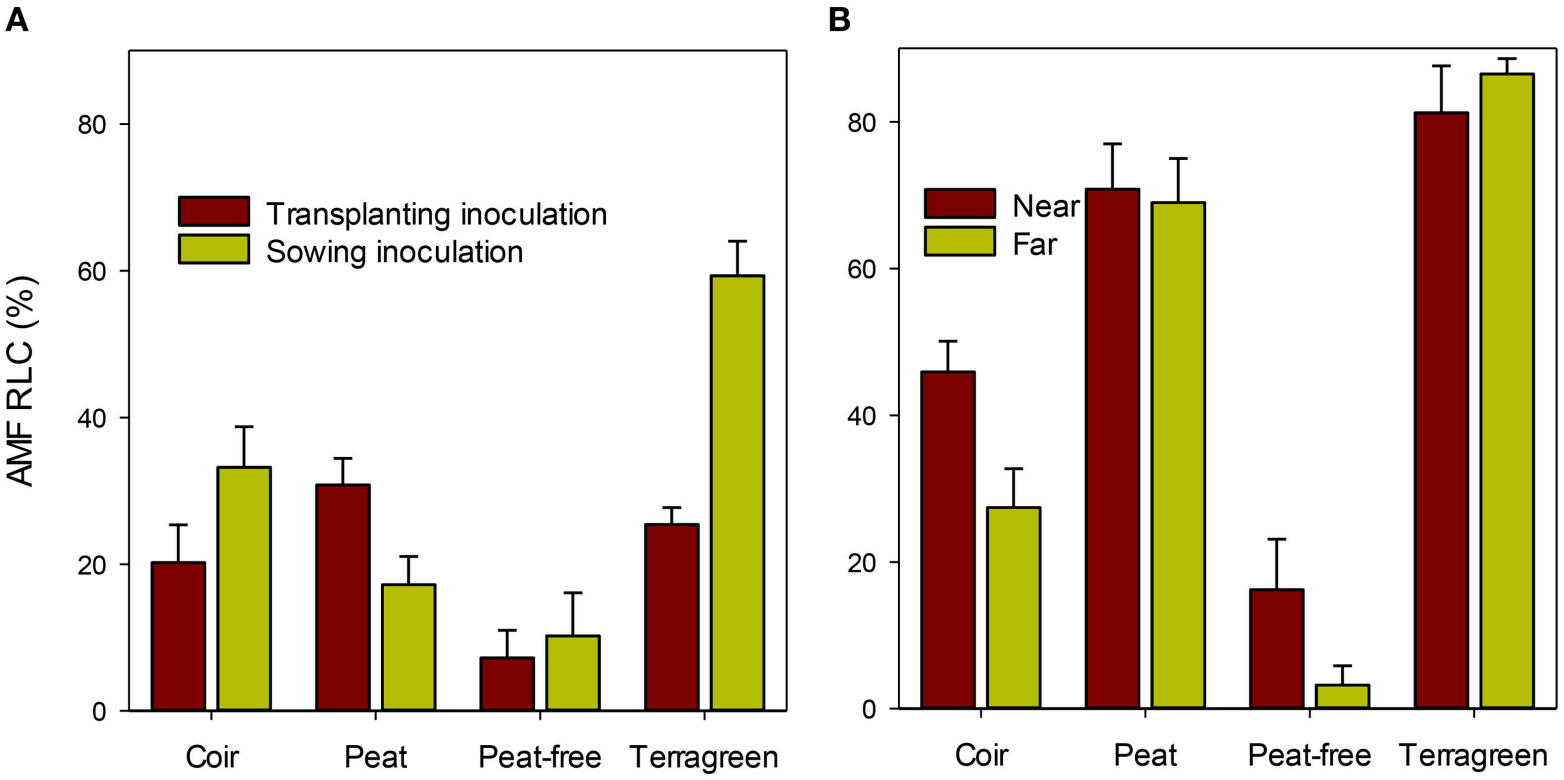
**Average root length colonization (RLC) by AMF of maize roots in four different substrates at two inoculation times (either during the sowing or during the transplanting) when assessed at 4 weeks (A) [sed for the substrate means = 7.75%]; average RLC at the two root positions relative to the inoculation site when assessed at 10 weeks (B) [sed is 6.7 and 4.9% for near to and away from inoculation sites, respectively]**.

At 4 weeks after transplanting, RLC did not differ significantly between plants inoculated twice at both pre- and post-emergence and those plants only inoculated once (24.4% [both] vs. 25.5% [single]; Table 2). In contrast, inoculation during sowing resulted in greater [*F*_(1, 43)_ = 5.5, *P* < 0.05] RLC than inoculation during transplanting: 30.0 vs. 20.9%. There were large [*F*_(3, 43)_ = 19.6, *P* < 0.001] differences in RLC between four substrates, accounting for ca. 44% of the total variation. Average RLC for Terragreen was 40.9%, significantly (*P* < 0.01) greater than coir (25.5%), and peat (27.1%), which in turn was greater (*P* < 0.001) than the peat-free substrate (6.8%). In addition, RLC differences between two inoculation timings varied with substrates [*F*_(3, 43)_ = 6.6, *P* < 0.001]: for both coir and Terragreen, inoculation in sowing led to greater RLC than during transplanting, which was opposite to the situation for peat (Figure 4), and the difference for peat-free was very small.

**Table 2:** **Summary of ANOVA (*F*-values) of AMF root length colonization (RLC) where granular AMF products were applied to maize plants grown in four different types of substrates at sowing time only (SO), or transplanting time only (TO), or both (ST)**.

**Terms**	**Degree of freedom**	**RLC 4 weeks**	**RLC-near 10 weeks**	**RLC-away 10 weeks**
Block	4	1.17	2.94	1.58
AMF inoculation time	2	2.80	0.95	1.51
SO vs. TO	1	0.10	0.95	2.37
ST vs. (SO + TO)	1	5.50^*^	0.94	0.64
Substrate	3	19.6^***^	27.9^***^	91.7^***^
AMF inoculation × Substrate	6	3.84^**^	1.24	1.79
(SO vs. TO) × Substrate	3	1.11	0.73	2.25
[ST vs. (SO + TO)]× Substrate	3	6.56^***^	1.76	1.33
Residual	43^+^			

AMF colonization was assessed at 4 and 10 weeks post-transplanting; for the 10 week assessment, two types of roots were sampled—those near or further away from the AMF inoculation site.

*, **, *** Significant at the level of 0.05, 0.01, and 0.001, respectively.

+ For RLC near the inoculation sites, there was only 42° of freedom for the residuals.

When assessed 10 weeks after transplanting, the only significant difference in RLC was related to the four substrates [Table 2; *F*_(3, 42)_ = 27.9 and *F*_(3, 43)_ = 91.7 (*P* < 0.001)] for RLC near and further away from the inoculation site, respectively. For roots near the inoculation site, RLC was greatest for Terragreen (81.2%) and least for peat-free (16.2%; Figure 4B) and RLC did not differ significantly between Terragreen (81.2%) and peat (70.8%). The relative differences in RLC in roots further away from the inoculation site between the four substrates were similar as for the near-inoculation-site, except that the difference between Terragreen and peat was significant (*P* < 0.05). RLC differences between the two root positions also varied [*F*_(1, 42)_ = 4.5, *P* < 0.01; Table 2] with substrates: for both coir and peat-free, RLC was less on the roots far from the inoculation site whereas no such differences were observed for Terragreen and peat (Figure 4B).

#### Effect of coir substrate on AMF colonization

Overall AMF colonization was lower [*F*_(1, 23)_ = 5.6, *P* < 0.05] in coir (13.1%) than in Terragreen (29.3%); average colonization was greater [*F*_(1, 23)_ = 10.5, *P* < 0.01] in maize (36.8%) than in strawberry (13.4%). There were no significant interactions between hosts and substrates in affecting AMF colonization. Although, there were significant differences between treatments, the level of AMF colonization varied considerably within individual treatments.

**Figure 5:**
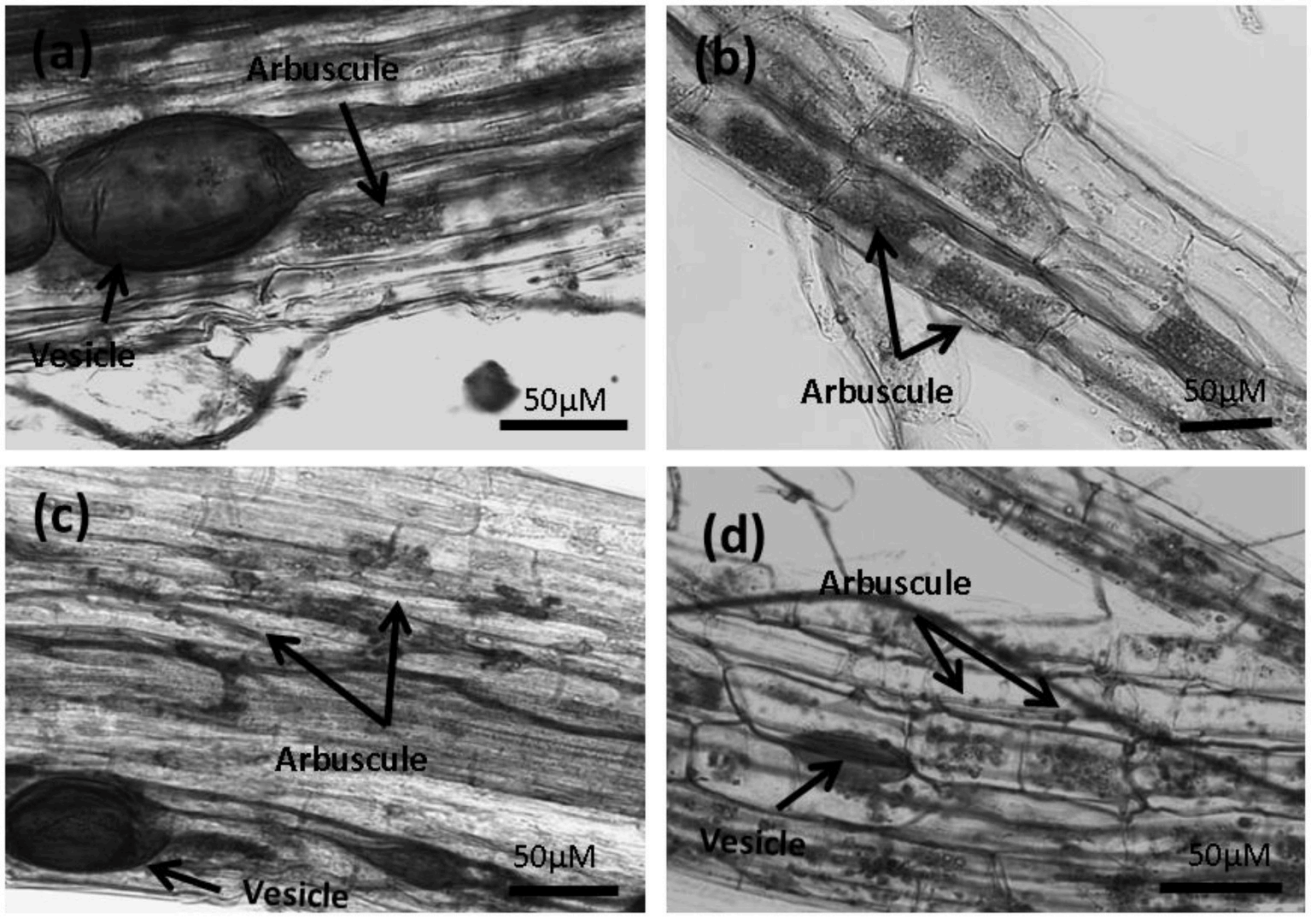
**Micrographs of strawberry roots stained using trypan blue showing mycorrhizal structures**. **(A,B)** Plants grown in Terragreen substrate, and **(C,D)** plants grown in coir substrate. Differences can be seen between the arbuscule formation and development in the coir substrate.

The morphology of the mycorrhizal structures in coir was different from those in Terragreen (Figure 5), in which normal colonization by AMF with fully-formed clear arbuscule structures was observed. Because of the changed structure in coir, a much larger root sample was assessed for colonization using a grid line technique. In strawberries growing in coir, the arbuscules and vesicles were small, underdeveloped, and their presence was inconsistent in the colonized roots—in many cases only hyphae were observed.

## Discussion

Inoculation with a commercial AMF product in coir increased yield and number of class I fruit of strawberry, particularly under stress conditions of deficient irrigation or low nitrogen input. The granular product of mixed AMF species resulted in a consistent limited benefit (though not statistically significant) to strawberry plants than the liquid inoculum, although the same number of infective propagules was added at planting in either formulation. This difference could be because the liquid formation has only a single species (*R. irregularis*), whereas the granular has four species of AMF. Thus, there could be synergistic interactions among AMF species in promoting plant growth (Wagg et al., 2011). However, recent work suggested that addition of two AMF species/strains did not result in improved performance of strawberry plants in compost or Terragreen relative to the use of individual species/strains (Robinson-Boyer et al., 2015). Another possible cause could be that with the liquid formulation there may be considerable losses of inoculum with irrigation water to the bottom of the bag that was not reachable by roots. The increase in fruit production was mainly associated with either of the two stress conditions singly, but not when combined. This suggests that AMF can alleviate the negative effects of either drought or low nitrogen. However, the positive effects of AMF on plant may be limited if plants are subjected extreme or combined stresses, which needs further research. Currently, we are conducting transcriptomics research trying to shed light on this AMF-strawberry interaction in coir.

Surprisingly, with such an appreciable increase in plant growth/yield, there were very low levels of root colonization by AMF under any of the conditions in coir. This is remarkable given that the original native colonization found in the planting material (runners lifted from field-grown mother plants) had ca. 20% RLC by AMF, which did not appear to spread and establish into the roots produced post-planting in the control plants. The detection of AMF was by root staining and thus does not give an indication of the viability of the AMF colonization present. This may be important considering these runner plants would have overwintered at −2°C prior to planting. Root colonization by AMF in the treated plants was also mostly found in roots that had developed in close proximity to the inoculation site. Reduced root colonization by AMF in coir was also shown for maize, normally recognized for high RLC levels of AMF. Here we clearly show that the level of root colonization in maize was affected by the substrate in which the plant was grown and was significantly reduced when grown in coir. Again the colonization detected in maize grown in coir was largely close to the site of inoculation.

Such low colonization of roots in coir could indicate that (1) coir is a harsh environment for mycorrhiza to colonize roots, (2) movement of inoculum in coir is limited (as irrigation is well controlled to prevent run-off), (3) spore production from colonized roots is limited and (4) changes to plant root physiology in substrate. In addition to the low level of colonization by AMF in coir, the AMF structures appear to be more compact and immature in coir than in soil and Terragreen on both maize and strawberry. This compact AMF structure in coir was also observed in clover (data not shown). Studies (Isayenkov et al., 2004; Hart et al., 2013) have shown that the level of colonization of a root is not necessarily an indicator of mycorrhizal benefit; however it is notable to record such low levels providing large and consistent plant growth promoting effects. It is possible that in a substrate environment, which is highly artificial for plant growth containing no background level of beneficial micro-organisms, colonization by AMF, even at a low level, may be highly beneficial for plant production.

Another consideration in applying AMF in commercial agriculture is to what extent there is a specific interaction of plant growth environment with AMF species or strain genotype. Multiple variants of sequences have been shown to occur within individual spores and isolates, as well as within and between species of the Glomeromycota (Rodriguez et al., 2001). It is important to determine how different growing environments and host plants could influence AMF genomic changes (Krüger et al., 2015) and consequently their beneficial effects on plant growth, enabling specific AMF products under specific conditions to maximize their beneficial effects. Further research is needed to investigate the inter-relationship of AMF effect, colonization structure and colonization levels in different types of substrates.

It is known that in other crop plants, e.g., wheat, genotypes and cultivars can differ in the extent to which they form an association with AMF (Al-Karaki and Al-Raddad, 1997). Further work is needed to assess to what extent the benefit associated with AMF inoculation in coir is dependent on strawberry genotypes (cultivars). To fully exploit the positive effect of AMF on strawberry in coir substrate, further work is needed to clarify to what extent the ability of specific strawberry genotypes being colonized by AMF in coir is heritable. If this trait is controlled genetically, this could be exploited to breed strawberry plants that can be easily colonized by AMF in substrate to increase their cropping potential and tolerance to pathogens, e.g., powdery mildew and *Phytophthora* diseases.

In conclusion this work demonstrates that there is a role for AMF in the commercial production of strawberry when grown in substrate and they could be a valuable tool for sustainable cropping of this important fruit crop especially under low-input productions systems. Current levels of high intensity agriculture are no longer sustainable primarily due to energy costs of N fertilizers and the decreasing supplies of P (Cordell et al., 2009), along with a decreasing armory of pesticides (due to legislation) and water limitation. Further studies such as this are needed to improve our knowledge of how best to apply and use these beneficial organisms to successfully incorporate them into sustainable commercial cropping systems for soft fruit and other commercial crops. With a greater understanding of the application and benefits of these beneficial microbes there is a real possibility for their use in aiding sustainable crop production.

## Author contributions

LR, is the lead author, and undertook the bulk of project management, practical work and writing. WF contributed largely to the metagenomics studies undertaking practical work and writing. NG, KH worked on inoculum preparation and the strawberry experiments with practical work, data collection, analysis, and writing. RH contributed to the metagenomics analysis and writing. PJ was a PI on the metagenomics work, contributed to analysis, interpretation and writing. XX was PI on all the work, contributing to analysis, metagenomics analysis, interpretation, and writing.

### Conflict of interest statement

The authors declare that the research was conducted in the absence of any commercial or financial relationships that could be construed as a potential conflict of interest.
